# Assessment of Low‐Frequency Magnetic Fields Emitted by DC Fast Charging Columns

**DOI:** 10.1002/bem.22254

**Published:** 2020-02-11

**Authors:** Germana Trentadue, Rosanna Pinto, Marco Salvetti, Marco Zanni, Konstantinos Pliakostathis, Harald Scholz, Giorgio Martini

**Affiliations:** ^1^ European Commission Joint Research Center (JRC) Ispra Italy; ^2^ ENEA—Italian National Agency for New Technologies Energy and Sustainable Economic Development RC Casaccia Rome Italy

**Keywords:** DC charging, fast charging, human electromagnetic field exposure, magnetic flux density, e‐mobility

## Abstract

The expected imminent widespread use of electromobility in transport systems draws attention to the possible effects of human exposure to magnetic fields generated inside electric vehicles and during their recharge. The current trend is to increase the capacity of the battery inside the vehicles to extend the available driving range and to increase the power of recharging columns to reduce the time required for a full recharge. This leads to higher currents and potentially stronger magnetic fields. The Interoperability Center of the Joint Research Center started an experimental activity focused on the assessment of low‐frequency magnetic fields emitted by five fast‐charging devices available on the market in recharge and standby conditions. The aim of this study was to contribute to the development of a standard measurement procedure for the assessment of magnetic fields emitted by direct current charging columns. The spectrum and amplitudes of the magnetic field, as well as exposure indices according to guidelines for the general public and occupational exposure, were recorded by means of a magnetic field probe analyzer. The worst‐case scenario for instantaneous physical direct and indirect effects was identified. Measurements within the frequency range of 25 Hz–2 kHz revealed localized magnetic flux density peaks above 100 μT at the 50 Hz frequency in three out of five chargers, registered in close proximity during the recharge. Beyond this distance, exposure indices were recorded showing values below 50% of reference levels. Bioelectromagnetics. 2020;41:308–317 © 2020 The Authors. *Bioelectromagnetics* published by Wiley Periodicals, Inc.

## INTRODUCTION

This study specifically focuses on the assessment of low‐frequency magnetic fields emitted by devices used for the fast charging of electric vehicles (EVs). The Joint Research Center (JRC) conducted a measurement campaign on five direct current (DC) fast chargers in standby condition and during the quick recharge (up to 50 kW). Measured values of the magnetic flux density (B‐field) were compared with reference levels (RLs) and action levels set out, respectively, for general public exposure with the Council Recommendation 1999/519/EC [EC, 1999] and for workers' exposure with the 2013/35/EU directive [EU, 2013]. The European legislative framework recommends compliance with guidelines published by the International Commission on Non‐ionizing Radiation Protection (ICNIRP) for limiting exposure based on adverse health effects reported in the available scientific literature. The Council Recommendation is based on ICNIRP guidelines for time‐varying electric, magnetic, and electromagnetic fields (EMFs) up to 300 GHz [ICNIRP, 1998], while the Directive refers to more recent guidelines for static magnetic fields [ICNIRP, 2009], and for time‐varying electric and magnetic fields [ICNIRP, 2010]. Guidelines indicate restrictions according to acute effects with established evidence: electrostimulation of nerves at low frequencies (1 Hz–10 MHz) and the heating of body tissues at higher frequencies (100 kHz–300 GHz).

Biological effects related to chronic exposure to EMFs have been reviewed by several international bodies that agree with the World Health Organization in recognizing that the scientific evidence could not confirm any correlation between exposure and long‐term adverse health effects [WHO, 2007; Repacholi, 2012; Samaras et al., 2015]. The International Agency for Research on Cancer (IARC) classified extremely low‐frequency (ELF) magnetic fields as possibly carcinogenic in 2002, due also to limited evidence that ELF magnetic fields might be a risk factor for childhood leukemia [IARC, 2002].

On the contrary, low‐frequency magnetic fields may represent potential electromagnetic compatibility (EMC) threats to equipment such as passive medical devices that are worn on the body or active implanted medical devices (AIMD) [Ruddle et al., 2015]. EMC requirements for AIMD (e.g., EN 45502‐2‐1) are based on RLs identified in ICNIRP [1998] guidelines despite the fact that ICNIRP guidelines consider possible interference with medical devices beyond their scope.

The scientific literature includes some studies regarding the electromagnetic environment inside conventional, hybrid, and EVs [Moreno‐Torres et al., 2013; Karabetsos et al., 2014; Hareuveny et al., 2015; Vassilev et al., 2015; Tell and Kavet, 2016]. The issue of human exposure to EMFs emitted during EV recharge with wireless power transfer systems is specifically addressed in the publicly available specification of the International Standard Organization ISO/PAS 19363 regarding “Electrically propelled road vehicles—Magnetic field wireless power transfer safety and interoperability requirements” [ISO, 2017]. Other scientific studies related to this topic have already been published [Carlson and Normann, 2014; Campi et al., 2019]. Furthermore, to the authors' knowledge, there is no evidence in the literature about studies concerning exposure assessment to EMFs emitted by conductive charging stations. According to the Society of Automotive Engineers (SAE), power levels at stake and the time required for the recharge vary widely depending on the technology (level 1: 1.5–3 kW, level 2: 10–20 kW, DC levels > 40 kW). The main concern about fast chargers (charging powers > 40 kW) is related to the high currents used to speed up the charging process and consequently potential exposure to low‐frequency magnetic fields. The reference standard of the International Electro‐technical Commission (IEC) 61851–23:2014, “Electric vehicle conductive charging system—Part 23: DC electric vehicle charging station,” does not mention any method for evaluating magnetic field emissions, despite the high powers involved during fast charging. The aim of this study was to devise a standard, reproducible, and representative method for the assessment of magnetic fields emitted by DC fast chargers.

## MATERIALS AND METHODS

The test was conducted by means of a low‐frequency electric and magnetic isotropic tri‐axial field probe (EHP50E; Narda Safety Test Solutions, Cisano sul Neva, Italy), which is designed according to the requirements of the IEC 61786‐1 standard [IEC, 2013] for measuring instruments. The declared anisotropy for the magnetic probe is 1.4% (0.12 dB) for a cubic case of 1,000 cm^3^ circa and the declared resolution is 1 nT. It is capable of performing measurements both in the frequency domain and in the time domain. When the probe is used for spectral measurements, the samples are collected and processed by fast Fourier transform. Its internal software automatically calculates the total integration of the field over the selected frequency range and compares the result with the threshold set by the 1999/519/EC recommendation [EC, 1999], assessing whether this is exceeded. The exposure index is calculated according to the following formula:

(1) $$\sum_{j=1\;Hz}^{10\;MHz}\frac{X_{j}}{AL(X_{j})}\leq 1$$

where *X_j_* is the field strength at frequency *j*, and *AL*(*X_j_*) is the field strength RL at frequency *j* [ICNIRP, 1998]. This formula applies to each Cartesian component of the field considering harmonics additive in their effects while neglecting the phase of the waveforms. This could lead to conservative and false‐positive results with complex waveforms like the ones emitted by fast chargers (see Appendix). For this reason, the analyzer provides a full‐time domain approach, exactly emulating the transfer functions of hardware filters suggested by the guidelines with the weighted peak method (WPM) [ICNIRP, 2003]. The WPM consists of weighting the complex waveform with a filter function, which is related to the basic restriction or RL to take into account both the amplitude and phase of the spectral components. This filtering can be presented mathematically as

(2) $$\left|\sum_{i}\frac{A_{i}}{EL_{i}}\;Cos(2\pi f_{i}t+\theta_{i}+\varphi_{i})\leq 1\right|$$

In Equation (2), *t* is time, *A_i_* is the amplitude of the *i*th harmonic component of the field, *EL_i_* is the exposure limit at the *i*th harmonic frequency *f_i_*, and *θ*_*i*_, $\varphi_{i}$ are phase angles of the field and phase angles of the filter at the harmonic frequencies, as indicated in ICNIRP [2010]. Equations (1) and (2) represent the calculation of the exposure indices according to the two different methods. In both cases, the calculation includes the ratio between the observed exposure and the limit value. However, while Directive 2013/35/EU [EU, 2013] recommends the WPM as the reference assessment method for non‐thermal effects in the frequency range 1 Hz–10 MHz to avoid overestimation, the Council 1999/519/EC recommendation [EC, 1999] for general public exposure does not include this approach.

Five DC fast charging columns, available on the market, were tested inside the Interoperability Laboratory of the Joint Research Center (Ispra, Italy). The chargers were equipped with combined charging system and CHAdeMO charging options and they were connected to the JRC electricity grid providing 400 V AC by means of a max 125 A, 50 Hz wall socket [Trentadue et al., 2018]. Table 1 describes the main technical specifications of the devices under test.

**Table 1:** Chargers’ Main Specifications [Trentadue et al., 2018]

Charger	Electrical specifications	Efficiency and power factor	Weight and noise
A	400 V_ac_; 200–500 V_dc_; 20–44 kW_dc_; 20–43 kW_ac_; Max 63 A_ac_/125 A_dc_	Not available	350 kg and <55 dB
B	400 V_ac_; 300 Arms; 120 kW_dc_; 65 kW_ac_	96% and 0.99	400 kg and 60 dB
C	400 V_ac_; 80 A; 55 kVA; 50 kW_dc_; Max 120 A; standby power: 100 W (w/o heater); 1,000 W	>92% and not available	400 kg and <45 dB
D	400 V_ac_; 73 A_ac_, 43 kVA; Max 120 A_dc_	>93% and 0.98	600 kg and <55 dB
E	400 V_ac_; 80 A; standby power: 250 W (w/o heater); 1,000 W	96% and not available	350 kg and <60 dB

A full characterization of the space around the chargers was accomplished, assessing the background environment as well. Background measurements were performed for both electric and magnetic fields. Electric fields are very easily perturbed by humidity variations and by the presence of conductive objects, such as poor electrical conductors, buildings, trees, and people. A minimum distance of 2 m between the operator and the probe is required [IEC, 2014], leading to challenges and difficulties in conducting measurements. On the contrary, magnetic fields have difficulty being shielded at low frequency. For the above reasons and because the involved voltages were always below 1,000 V, the produced electric field was assumed to be far below the RLs reported in the 1999/519/EC recommendation [EC, 1999]. The test campaign focused only on magnetic fields which were considered more relevant for low and intermediate frequency exposure.

**Figure 1:**
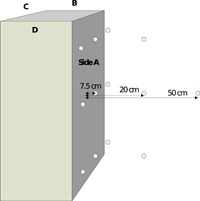
Grid distribution of measurement points.

The measurements were conducted in two different operating conditions: standby and during the recharge at full load. To capture the worst‐case scenario with high currents and high values of B‐field, measurements were performed within a time interval during which the delivered power is stable and close to 50 kW. This condition was reached during a recharge between 10% and 50% of the state of charge, depending on the vehicle. The vehicle was placed at a distance of 1 m from the charging column. The adopted strategy foresaw a frequency domain assessment, which searched for maximum magnetic field points or “hot spot” identification as a rapid scan of the device under test (DUT) and a spatial assessment of the spectrum with a fixed maximum threshold at a fixed distance. This measurement methodology was necessary to detect direct and indirect effects with a possible instantaneous impact on human health, such as interference with electronic medical devices. Field strengths above the RLs at the position of the device or its sensing leads (when present) may result in a malfunction, which would represent a risk to people wearing them. In such a case, the spatial average foreseen in the IEC 61786‐2 standard (see Appendix) would not be an effective constraint, while the proposed approach, albeit conservative, would be more adequate. Therefore, a grid of measurement points was defined to take into account the spatial non‐uniformity of the field and to simulate a possible exposure situation for generic users, workers, and any person located within the vicinity of the charging column. Measurement points were distributed according to the geometry of the charger's shell, with more being placed in close proximity of the charger. The measurements were performed on each side of the charging column at three different distances between the surface under test and the center of the probe (7.5, 20, and 50 cm). The number of measurement points varied with the size of the column and detected field levels. Thus, at a distance of 7.5 cm, more linearly distributed points were considered, while fewer points were considered for greater distances where the field intensity decreased. Figure 1 shows an example of the measurement grid that was applied to each side of the tested charger.

The set frequency range (from 25 Hz up to 2 kHz) was considered a good compromise between the limitations of the available probe and range of interest for the measurement for both operating conditions (standby and recharge at full load). A first rapid scan of the DUT was performed with the probe placed at a minimum distance of 7.5 cm from the charger. Although these measurements were not representative of the calculated average over the human body because the distance between probe and DUT was too short, they revealed the maximum values of B‐field that a generic user could experience during fast charging.

## RESULTS

For each charger, background measurements of magnetic and electric fields were performed with the DUT switched off. Recorded wideband values of B‐field did not exceed 40 nT while the electric field showed values below 0.6 V/m.

**Figure 2:**
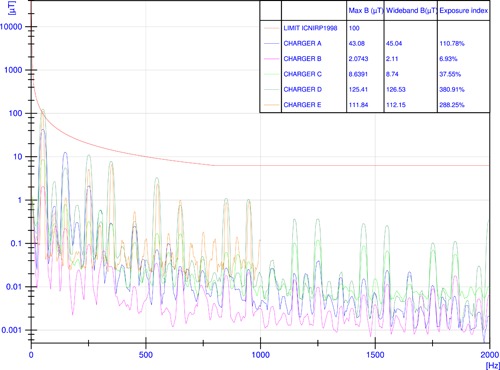
Magnetic flux density *B* in the frequency domain recorded at a distance of 7.5 cm from the maximum emission point during a recharge at a power between 40 and 50 kW circa within the frequency range (25 Hz–2 kHz) and related exposure indices according to ICNIRP [1998] guidelines (chargers A–E).

In all chargers' assessment in the frequency domain, the highest peaks of B‐field were recorded at a frequency of 50 Hz followed by the harmonics of this fundamental frequency, confirming that the predominant magnetic field waveform for these devices was the sinusoidal waveform at the power frequency. Depending on the charger, the harmonic content was very different as well as the point where the highest peaks of the magnetic field were recorded. This was mainly due to the internal design of each charger, but it was also related to the materials used for the outer shell. Figure 2 shows the B‐field recorded during the recharge at full load at a distance of 7.5 cm between the maximum emission point and the center of the probe. The intensity of the 50 Hz harmonic reached values above the ICNIRP [1998] RLs for the general public in two of five chargers (namely, chargers D and E). For the whole spectrum (25 Hz–2 kHz), the results did not satisfy the multiple frequency calculation (Equation 1) for three out of five chargers (A, D, and E). The exposure index (Equation 1), automatically calculated by the instrument's software and expressed in percentage, reached a maximum value of 381% for charger D, followed by charger E with 288%, and charger A with 111%.

**Figure 3:**
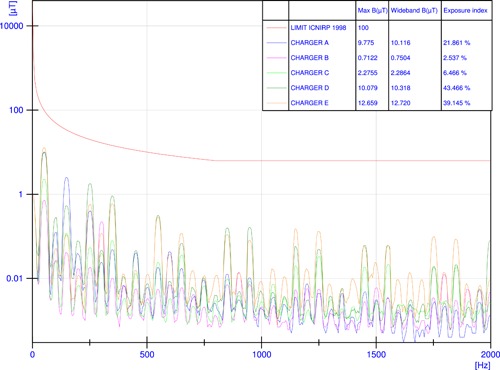
Magnetic flux density *B* in the frequency domain recorded at a distance of 20 cm from the maximum emission point during a recharge at a power between 40 and 50 kW circa within the frequency range (25 Hz–2 kHz) and related exposure indices according to ICNIRP [1998] guidelines (chargers A–E).

Figure 3 shows that B‐field peaks measured at a frequency of 50 Hz decrease accordingly to the position of the probe, reaching values around 10 μT in three out of six chargers (A, D, and E) at a distance of around 20 cm. Due to DUT availability constraints, only a spatial characterization was performed. The spatial characterization of chargers at a distance of around 20 cm shows values of B‐field far below the Council's 1999/519/EC [EC, 1999] recommended RLs, with values for the exposure indices (Equation 1) always below 50%. Table 2 reports the characterization of each side of the chargers at a distance of 50 cm in terms of the highest peak of the frequency spectra and the wideband value of B‐field during a recharge at a power between 40 and 50 kW circa. For each charger, the highest peaks occurred at a frequency of 50 Hz, confirming that the spectral content was concentrated at this frequency.

**Table 2:** Magnetic Flux Density *B* Peaks at 50 Hz and Wideband Value (25 Hz–2 kHz) Measured at a Distance of 50 cm From Different Sides of the Charger During a Recharge at a Power Between 40 and 50 kW circa

	Side A		Side B		Side C		Side D	
Charger	*B* (μT), 50 Hz	Wideband, *B* (μT)	B (μT), 50 Hz	Wideband, *B* (μT)	*B* (μT), 50 Hz	Wideband, *B* (μT)	*B* (μT), 50 Hz	Wideband, *B* (μT)
A	0.180	0.189	0.443	0.445	0.339	0.342	0.681	0.684
B	N.A.	N.A.	0.798	0.801	0.088	0.089	0.197	0.199
C	0.704	0.706	0.737	0.74	0.363	0.365	0.581	0.583
D	0.600	0.636	0.668	0.684	0.583	0.655	0.479	0.498
E	0.314	0.322	0.218	0.22	0.231	0.237	0.172	0.207

**Figure 4:**
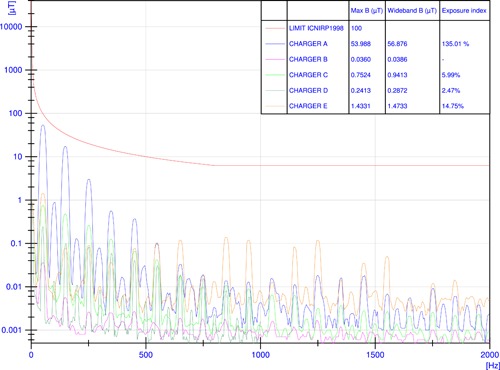
Magnetic flux density *B* in the frequency domain recorded at a distance of 7.5 cm from the maximum emission point in standby condition within the frequency range (25 Hz–2 kHz) and related exposure indices according to ICNIRP [1998] guidelines (chargers A–E).

In the standby condition, the B‐field was measured at the same worst‐case positions identified during the recharge at full load. Figure 4 shows the values of B‐field recorded in the frequency domain at a distance of 7.5 cm from the maximum emission points. Values of the maximum peaks at a frequency of 50 Hz, as well as wideband values and the exposure index according to Equation 1, are reported.

Table 3 reports the highest peaks of the frequency spectra and wideband value of B‐field measured for each side of the chargers' shell at a distance of 50 cm in standby condition. Figure 4 and Table 3 also show that the highest peaks occurred at a frequency of 50 Hz for chargers in standby.

**Table 3:** Magnetic Flux Density *B* Peaks at 50 Hz and Wideband Value (25 Hz–2 kHz) Measured at a Distance of 50 cm From Different Sides of the Charger in Standby Conditions

	Side A		Side B		Side C		Side D	
Charger	*B* (μT), 50 Hz	Wideband, *B* (μT)	*B* (μT), 50 Hz	Wideband, *B* (μT)	*B* (μT), 50 Hz	Wideband, *B* (μT)	*B* (μT), 50 Hz	Wideband, *B* (μT)
A	0.3891	0.4343	0.1653	0.1729	0.1465	0.1517	0.1602	0.1684
B	0.0302	0.0326	0.0424	0.045	0.0382	0.0405	0.0448	0.047
C	0.0304	0.0341	0.0558	0.0622	0.0364	0.0385	0.0388	0.0409
D	0.6001	0.6357	0.6679	0.6841	0.5834	0.6553	0.4791	0.4982
E	N.A.	N.A.	0.0349	0.0368	0.0356	0.0378	0.0318	0.033

At the time of the test campaign, the probe for static magnetic fields was not available. Nevertheless, the resulting stray magnetic field produced by the DC current along the charging cable was considered to be far below the RL reported in the 1999/519/EC recommendation [EC, 1999], given the cabling architecture.

Spot measurements with the WPM were performed, especially at points of the maximum magnetic field. The results, reported by the instrumentation's software as a percentage, refer to Equation (2) and to RLs of ICNIRP [2010] guidelines for general public and for workers, which are more relaxed compared with the RLs set in the 1999/519/EC recommendation [EC, 1999] for the general public at frequencies below 100 kHz, due to increased knowledge of electrostimulation phenomena and its associated dosimetry. Table 4 reports the results obtained with the WPM in the frequency range 1 Hz–100 kHz measured at a distance of 7.5 cm from the maximum emission point during a recharge at a power between 40 and 50 kW circa for chargers D–E. The highest value obtained with WPM was 17%, far below the values obtained with the multiple frequency method. Further studies would include measurements with WPM to evaluate compliance with the RLs of the 1999/519/EC recommendation [EC, 1999] over a broader range of frequencies in fixed points considered representative after the “hot spots” assessment.

**Table 4:** Weighted Peak (1 Hz–100 kHz) Measured at a Distance of 7.5 cm From the Maximum Emission Point During a Recharge at a Power Between 40 and 50 kW Circa (Chargers D–E) According to ICNIRP (2010) General Public and Occupational Guidelines

	Weighted peak (Equation 2)	
Charger	Population	Workers
D	50%	10%
E	72%	17%

## CONCLUSION

A reproducible measurement procedure was developed as an approach for the assessment of magnetic fields emitted during fast charging. Our measurement protocol takes into account the worst‐case scenario for instantaneous physical direct and indirect effects, particularly in relation to vulnerable users (electronic medical devices carriers, etc.). It also provides a robust test procedure for general public exposure. Indeed, the identification of the point of maximum field and its comparison with a fixed maximum threshold at a fixed distance is proposed to be effective in avoiding potential EMC threats to medical equipment that can happen instantaneously. The spatial assessment of the emitted spectrum takes into account spatial non‐uniformity. Measurements with WPM executed at fixed points, considered representative after the “hot spots” assessment, take into account temporal variations over an extended time interval, and they could be useful for determining if RLs of the 1999/519/EC recommendation [EC, 1999] are not exceeded over a broader frequency range.

All the chargers were tested according to the principles of measurements for a magnetic field defined in the IEC 62110 and IEC 61786‐2 standards. The B‐field values measured at a distance of 20 cm were far below the RLs of the 1999/519/EC recommendation [EC, 1999] for the general public, and consequently with the more relaxed threshold values of the 2013/35/EU directive [EU, 2013] for workers. In terms of general public exposure, although the tests were carried out on a limited sample of early fast charger models, the fact that in general the measured magnetic fields were below the RLs should increase confidence in the use of electromobility, providing an answer to growing and very often unjustified concerns from users. With reference to the 1999/519/EC recommendation [EC, 1999], the results at a shorter distance of 7.5 cm revealed that the B‐field had peaks above the RL at a frequency of 50 Hz, and did not satisfy Equation (1) between 25 Hz and 2 kHz. Such proximity would most likely occur with a user's physical contact with the charger. Therefore, these findings should be investigated and discussed more deeply, taking into account the conservative approach of the 1999/519/EC recommendation [EC, 1999]. In addition, these preliminary investigations and data are valuable for future consideration, for designing guidelines and for the development of a dedicated standard for EMF exposure during fast charging, as this technology becomes more widespread.

Spatial averages and temporal monitoring with combined use of dosimeters may be appropriate for the development of a measurement protocol for full characterization of the electromagnetic environment and for exposure assessment. Considering possible future scenarios, workers and/or ordinary users may be in recharge stations with hundreds of fast chargers. Hence, spatial and temporal combinations of fields should also be evaluated over an extended time frame in order to detect any peak during prolonged exposure. New legislative developments are expected soon. Associated technologies for battery charging such as DC fast charging columns (Mode 4, 50 kW) are continuously and rapidly evolving. A new generation of high power charging (HPC) is about to enter the market with power levels up to 350 kW to reduce the recharging time and concern about limited driving range. Higher power requires less time required for a full recharge, but also higher voltage and higher variable currents can lead to possible intense and time‐varying fields. A more extensive test campaign has just begun on HPC prototypes currently available at the Interoperability Center in Ispra in cooperation with ENEA, the Italian National Agency for New Technologies, Energy and Sustainable Economic Development. Exposure scenarios related to vehicles will be the subject of further studies. Moreover, tests in real outdoor conditions are under discussion to increase knowledge and trust in a possible future scenario with a higher concentration of fast chargers, even in urban areas.

## Supporting information

Supporting information.

Supporting information.

## Figures and Tables

**Figure A1:**
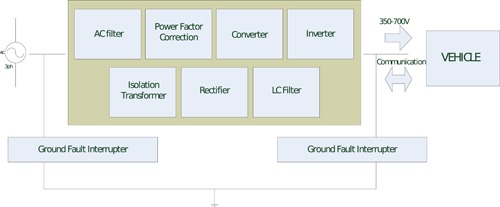
Block scheme of a DC quick charging power module.

## APPENDIX A

### Magnetic fields emitted during fast charging

Magnetic fields are produced by the load currents involved during fast charging. The load depends on several factors, such as user's activities, the strategy of the vehicle's battery management system, and outdoor temperature [IEC, 2014]. The delivered power during the charging process drastically increases during the first seconds after the charging connection. Then, it tends to stabilize at a power level, which depends on the above‐mentioned factors, before slowly decreasing.

Considering the typical block scheme of a DC fast charger (Fig. A1), the power electronics inside are in charge of the AC/DC conversion to provide, according to a dedicated communication with the vehicle to be charged, the appropriate DC charging power. Typically, an AC filter is installed to remove the negative impact of higher harmonic distortion. In the DC output lines, an LC filter is fitted to reduce ripple noises, which may cause lithium battery degradation [Anegawa, 2010]. The transformer, which separates the power grid and battery system to prevent accidental high voltage penetration, is a region where emitted magnetic fields can be more intense. Power lines and AC/DC converters generate discrete spectra characterized by a fundamental spectral component, the power frequency of 50 Hz, and other spectral content of harmonics occurring at integer multiples of the fundamental. At 50 Hz electric and magnetic fields are independent; the effects of the fields are accountable for small distances. After the AC/DC conversion, direct currents greater than 100 A flow through the charging cable. It contains two power wires, closely placed to each other, that carry currents directed in opposite directions toward positive and negative polarities of the high voltage battery inside the vehicle. This cabling architecture makes the resulting stray magnetic fields produced by the DC currents reduce each other on the outside of the cable until they reach the high voltage battery inside the vehicle.

In the specific case of charging systems, the emitted field is a superposition of sinusoidal fields of different frequencies where the power frequency of 50 Hz is the predominant spectral component. If the 50 Hz waveform had been purely sinusoidal, compliance with regard to RLs could have been assessed comparing the field value directly with the RL at that frequency. If such a comparison had been compliant, then compliance with the restrictions would have been ensured [Crotti and Giordano, 2009]. However, the real spectrum is a superposition of discrete and continuous broadband spectra, due to the presence of higher‐order harmonics of the fundamental frequency as well as transient phenomena, such as high peaks of currents once the charger is turned on, and intermittent fields with complex waveforms at higher frequencies emitted by the charger's other components, such as switching devices (usually insulated gate bipolar transistor), controllers, cooling fan, sensors, and routers.

Besides temporal variability, these sources should also be characterized in terms of spatial variability. In fact, magnetic fields emitted by DC chargers are spatially non‐uniform; thus the exposure within and outside the vehicle during a recharge may be strongly different. The user's and/or potential bystander's position, as well as the time spent close to the source, can considerably vary during the recharge. A possible user can move from a position close to the plug to one closer to the column, where the field is higher. Hence, the distance at which measurements are performed as well as the duration of the exposure are crucial parameters for the characterization. The IEC 62311 standard “Assessment of electronic and electrical equipment related to human exposure restrictions for electromagnetic fields (0 Hz–300 GHz)” [IEC, 2007] states that fields should be determined at the typical user position under normal operating conditions and then compared with product‐specific compliance criteria, which are still not addressed in IEC 61851 standards for conductive charging devices. Other standards such as IEC 61000‐3‐12 “Limits for harmonic currents produced by equipment connected to public low‐voltage systems with input current >16 A and ≤75 A per phase” [IEC, 2011] and technical reports such as IEC TR 61000‐2‐7 “Low‐frequency magnetic fields in various environments” [IEC, 1998] do not mention specific methods or considerations applicable at fast chargers. According to IEC 62110 (“Electric and magnetic field levels generated by AC power systems—Measurement procedures with regard to public exposure”) [IEC, 2009] and IEC 61786‐2 (“Measurement of DC magnetic, AC magnetic and AC electric fields from 1 Hz to 100 kHz with regard to exposure of human beings—Part 2: Basic standard for measurements”) standards [IEC, 2014], in the presence of non‐uniform fields, the exposure level should be calculated as the arithmetic mean between values measured at different positions at a horizontal distance of 0.2 m from the source's surface, where this default distance is the shortest distance required to calculate the average over the human body.
