# Supplementary figures and images for: Assessment of Low‐Frequency Magnetic Fields Emitted by DC Fast Charging Columns
Source: Bioelectromagnetics. 2020 Feb 11;41(4):308–17. doi: 10.1002/bem.22254 (PMC7217217; doi:10.1002/bem.22254)

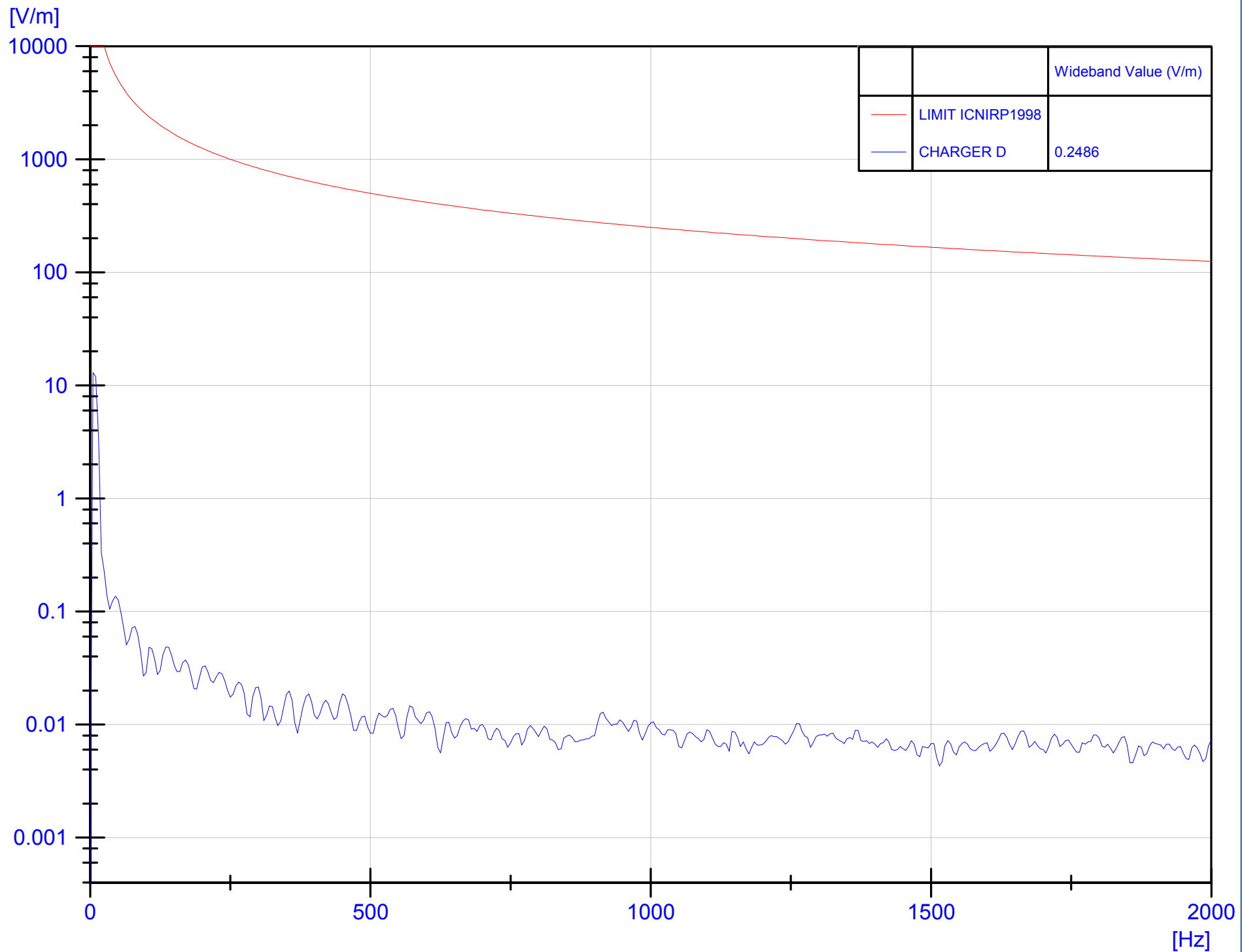

Supplement: Supplementary file 1 — Supporting information. [file BEM-41-308-s001.pdf]

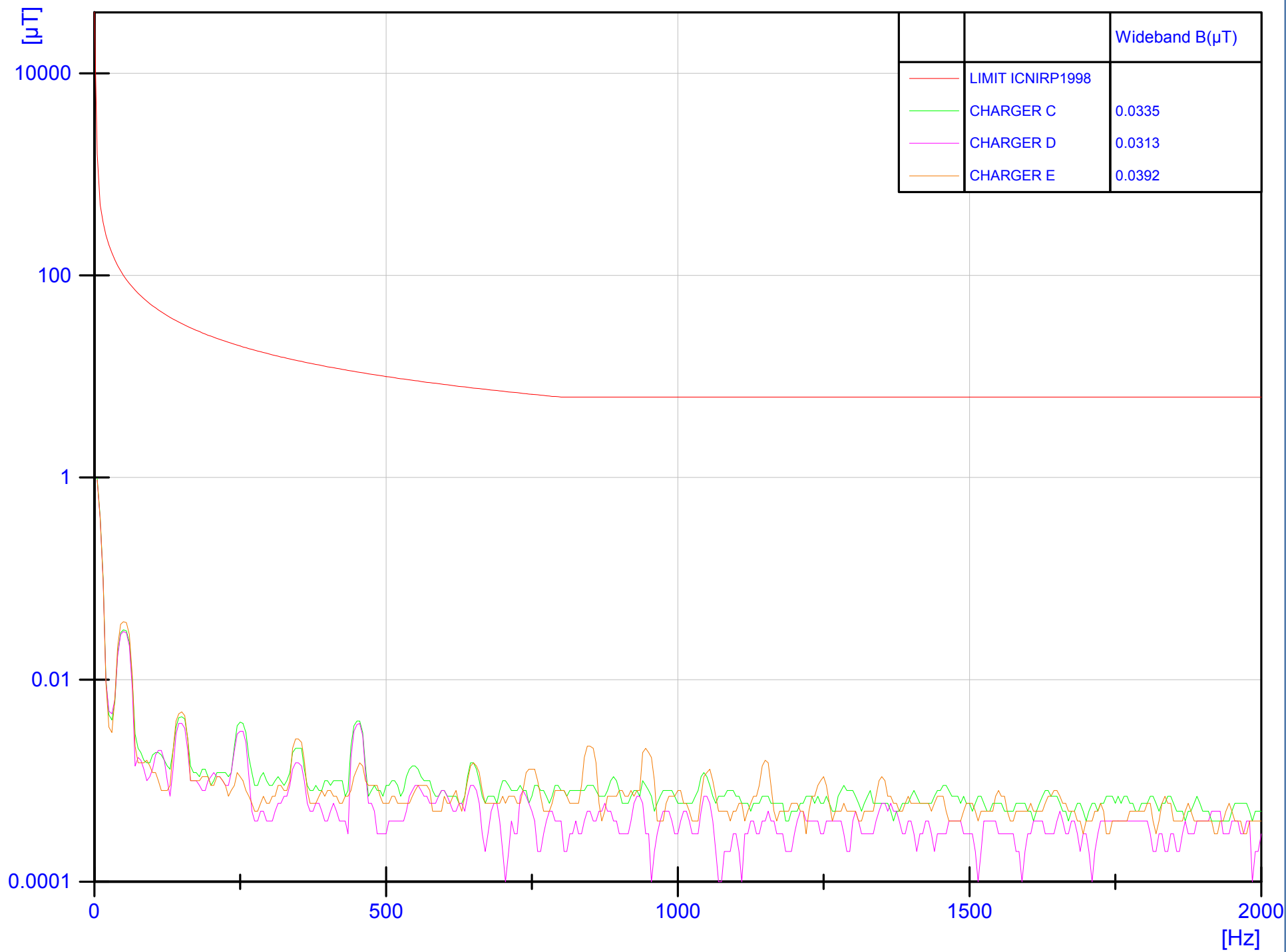

Supplement: Supplementary file 2 — Supporting information. [file BEM-41-308-s002.pdf]
